# *Lactobacillus helveticus* HY7801 Attenuates Inflammatory Edema and Vascular Leakage while Modulating Endothelial Barrier Responses

**DOI:** 10.4014/jmb.2608.08031

**Published:** 2026-09-18

**Authors:** Daehyeop Lee, Haeryn Jeong, Hyeonjun Gwon, Jimin Baik, Joo-Yun Kim, Jae-Jung Shim, Jae-Hwan Lee

**Affiliations:** R&BD Center, hy Co., Ltd., 22, Giheungdanji-ro 24beon-gil, Giheung-gu, Yongin-si 17086, Gyeonggi-do, Republic of Korea

**Keywords:** *Lactobacillus helveticus* HY7801, Paw edema, Endothelial integrity, Inflammatory responses, Extracellular vesicles

## Abstract

Tissue edema results from excessive interstitial fluid accumulation driven by inflammatory responses and increased vascular leakage. This study investigated the protective effects of *Lactobacillus helveticus* HY7801 (HY7801) against inflammatory activation, endothelial barrier dysfunction, and edema formation *in vitro* and *in vivo*. In lipopolysaccharide (LPS)-stimulated RAW 264.7 macrophages, HY7801 reduced nitrite accumulation and iNOS expression. In tumor necrosis factor-α (TNF-α)-stimulated human umbilical vein endothelial cells (HUVECs), HY7801 increased nitrite accumulation and eNOS expression, suppressed the expression of endothelial adhesion molecules (*ICAM1*, *VCAM1*, and *SELE*) and *CXCL8*, and enhanced the expression of the tight junction-associated genes (*TJP1* and *CLDN5*). Furthermore, HY7801 partially restored transendothelial electrical resistance (TEER), an indicator of endothelial barrier integrity, following TNF-α-induced impairment. In a λ-carrageenan-induced mouse paw edema model, HY7801 treatment attenuated paw swelling, histological tissue thickening, and vascular leakage. These effects were accompanied by increased junction-associated gene expression in inflamed paw tissue and reduced circulating inflammatory mediators. Based on these anti-inflammatory and endothelial barrier-protective effects, HY7801-derived extracellular vesicles (HY7801 EVs) were further evaluated *in vitro* as a candidate bioactive component of HY7801. HY7801 EVs enhanced *TJP1* and *CLDN5* expression while suppressing *ICAM1*, *VCAM1*, and *SELE* expression. Collectively, our findings suggest that HY7801 attenuates inflammation-associated tissue edema and vascular leakage through coordinated modulation of inflammatory and endothelial barrier responses, and support a potential role for HY7801 EVs as a bioactive component contributing to these effects.

## Introduction

The vascular endothelium forms a dynamic semipermeable barrier that regulates transvascular fluid and solute exchange and leukocyte trafficking, thereby maintaining vascular and tissue homeostasis [1, 2]. Endothelial barrier integrity is maintained through coordinated interactions between intercellular junctional complexes and the actin cytoskeleton, with junction-associated proteins including zonula occludens-1 (ZO-1) and claudin-5 contributing to the control of paracellular permeability [3, 4]. During acute inflammation, however, vasoactive and inflammatory mediators induce cytoskeletal remodeling and destabilize endothelial cell-cell junctions, resulting in endothelial hyperpermeability and the extravasation of plasma proteins and fluid into surrounding tissues [1, 5]. The resulting interstitial fluid accumulation is a major contributor to localized inflammatory edema [6, 7].

Endothelial barrier disruption can be further aggravated by inflammatory interactions between immune cells and the vascular endothelium [8]. Activated macrophages produce pro-inflammatory mediators and high levels of nitric oxide (NO) through inducible nitric oxide synthase (iNOS), contributing to inflammatory and nitrosative stress [9]. In contrast, endothelial nitric oxide synthase (eNOS)-derived NO supports vascular homeostasis, whereas inflammatory stimulation can impair eNOS-associated signaling and reduce endothelial NO bioavailability [10]. Inflammatory activation of endothelial cells also increases adhesion molecules and chemokines that promote leukocyte recruitment and transmigration [11, 12]. Together, these responses can amplify endothelial hyperpermeability and vascular leakage [3, 12], suggesting that suppression of inflammatory activation together with preservation of endothelial homeostatic responses may help limit inflammation-associated vascular disruption.

Nonsteroidal anti-inflammatory drugs (NSAIDs) are widely used to alleviate acute inflammation and edema, but high-dose or prolonged use is associated with gastrointestinal, renal, and cardiovascular adverse effects [13, 14]. Moreover, inflammation-associated vascular leakage involves not only inflammatory mediator production but also disruption of endothelial junctional and barrier-regulatory mechanisms [15, 16]. These considerations have prompted interest in complementary approaches that can attenuate inflammatory responses while supporting endothelial barrier integrity. Certain lactic acid bacteria (LAB) exhibit strain-specific immunomodulatory activities, and accumulating evidence suggests that selected LAB strains or their metabolites may also influence vascular endothelial function [17, 18]. In this context, *Lactobacillus helveticus* HY7801 (HY7801), isolated from healthy women, is of particular interest because previous studies have demonstrated its anti-inflammatory activity in distinct murine disease models, including reductions in pro-inflammatory cytokine levels and suppression of cyclooxygenase-2-, iNOS-, and nuclear factor κB-associated responses [19, 20]. However, whether HY7801 protects against endothelial barrier dysfunction and inflammation-associated vascular leakage remains unclear.

Beyond the effects of intact bacterial cells, LAB can release extracellular vesicles (EVs), nanosized membrane-enclosed particles that carry bioactive bacterial components and provide a potential cell-free route for bacterial-host communication [21]. Previous studies suggest that bacterial EVs can interact with host tissues beyond the site of bacterial residence and, under some conditions, may gain access to the systemic circulation [22]. HY7801-derived EVs have previously been characterized and shown to exert biological activity in host systems [23], supporting their evaluation as a candidate cell-free bioactive component of HY7801.

In the present study, we investigated whether HY7801 could attenuate inflammatory edema and vascular leakage while modulating inflammatory and endothelial barrier-associated responses. Using lipopolysaccharide (LPS)-stimulated macrophages and tumor necrosis factor-α (TNF-α)-stimulated endothelial cells, we examined the effects of HY7801 on NO-related responses, endothelial activation, and barrier regulation. The relevance of these responses was further examined in a λ-carrageenan-induced mouse paw edema model by assessing tissue swelling, vascular leakage, and junction-associated gene expression, and circulating inflammatory mediators. Finally, based on the previously reported biological activity of HY7801-derived EVs and the potential of bacterial EVs to mediate host responses beyond the gut, we examined whether HY7801 EVs could modulate endothelial inflammatory activation and junction-associated responses *in vitro*.

## Materials and Methods

### Preparation of *Lactobacillus helveticus* HY7801

*Lactobacillus helveticus* HY7801 (HY7801) was cultured in de Man, Rogosa, and Sharpe (MRS) broth (Difco, USA) at 37°C for 24 h. For *in vitro* experiments, the bacterial cells were harvested by centrifugation at 4,000 × g for 15 min at 4°C. The cell pellets were washed twice with sterile phosphate-buffered saline (PBS) and resuspended in PBS to obtain the target concentrations. The colony-forming units (CFU) were determined by serial dilution and plating on MRS agar. For animal study, HY7801 was administered as a freeze-dried product manufactured at the Probiotics Plant of hy Co., Ltd. (Republic of Korea). The viable cell count of the product was determined by serial dilution and plate counting and expressed as CFU/g. The daily amount administered to each animal was adjusted according to the viable count to achieve the designated dose.

### Cell Culture

RAW 264.7 macrophages and human umbilical vein endothelial cells (HUVECs) were obtained from the American Type Culture Collection (ATCC; USA). RAW 264.7 cells were cultured in Dulbecco’s Modified Eagle Medium (DMEM; Gibco, USA) supplemented with 10% fetal bovine serum (FBS) and 1% penicillin-streptomycin (P/S). HUVECs (passages 5−10) were cultured in endothelial cell medium (ECM; ScienCell Research Laboratories, USA) supplemented with 5% FBS, 1% P/S, and 1% endothelial cell growth supplement (ECGS). The culture medium was replaced with antibiotic-free medium prior to sample treatment. All cells were maintained at 37°C in a humidified atmosphere containing 5% CO_2_.

### Measurement of Nitric Oxide Production

RAW 264.7 cells were seeded in 96-well plates at a density of 1 × 10^5^ cells/well and pretreated with HY7801 at 1 × 10^6^ or 1 × 10^7^ CFU/mL for 4 h, followed by stimulation with 1 μg/mL LPS for 24 h. HUVECs were seeded in 96-well plates at a density of 1 × 10^5^ cells/well and pretreated with HY7801 at the same concentrations for 24 h. The cells were subsequently stimulated with 10 ng/mL TNF-α for 6 h. After incubation, the cell culture supernatants were collected, and nitrite accumulation was measured using the Griess reagent assay as an index of NO production. Absorbance was measured at 540 nm using a BioTek Synergy HTX Multimode Reader (Agilent Technologies, USA).

### Measurement of Transendothelial Electrical Resistance

HUVECs were seeded onto Transwell inserts (0.4 μm pore size; Corning Inc., USA) in 6-well plates at a density of 1 × 10^5^ cells/insert. The culture medium was replaced every 2-3 days until a confluent monolayer was formed. The cells were pretreated with HY7801 at 1 × 10^6^ or 1 × 10^7^ CFU/mL for 6 h, followed by stimulation with 10 ng/mL TNF-α for 18 h. Transendothelial electrical resistance (TEER) was measured using a MILLICELL-ERS system (Merck Millipore, USA) equipped with an MERSSTX01 electrode. TEER values were calculated by subtracting the resistance of a blank insert containing culture medium alone (R_blank_) from the resistance of the sample insert (R_sample_) and multiplying by the effective membrane area of the insert (4.69 cm^2^):

TEER (Ω·cm^2^) = (R_sample_ – R_blank_) × 4.69 cm^2^

### Animal Study Design

Male ICR mice (8 weeks old) were purchased from DooYeol Biotech Co., Ltd. (Republic of Korea). The mice were housed under controlled environmental conditions (19−25°C, 30−70% relative humidity, and a 12-h light/dark cycle) with *ad libitum* access to a standard diet and water. All experimental procedures were approved by the Institutional Animal Care and Use Committee (IACUC) of hy Co., Ltd. (Approval No. AEC-2026-0004-Y).

Following 1 week of acclimation, the mice were randomly assigned to five experimental groups (n = 12 per group): NOR, normal control; CRG, carrageenan control; DCF, carrageenan + diclofenac (10 mg/kg); HY7801L, carrageenan + HY7801 (1 × 10^8^ CFU/kg/day); HY7801H, carrageenan + HY7801 (1 × 10^9^ CFU/kg/day).

Mice in the HY7801L and HY7801H groups were orally administered HY7801 once daily for 3 weeks, whereas mice in the NOR, CRG, and DCF groups received an equivalent volume of saline during the same period. On the day of paw edema induction, the DCF group received a single intraperitoneal injection of diclofenac, whereas the other groups were injected with an equal volume of saline. One hour later, acute paw inflammation was induced in all groups except the NOR group by intraplantar injection of 50 μL of a 1% (w/v) λ-carrageenan (Sigma-Aldrich, USA) solution into the plantar region of the right hind paw. The NOR group was injected with an equal volume of PBS.

Within each group, eight mice were allocated to biochemical, molecular, and histopathological analyses, and the remaining four mice were allocated to the Evans blue extravasation assay. For the eight mice allocated to these analyses, paw thickness was measured up to 6 h after intraplantar injection. Immediately after the 6 h paw thickness measurement, the mice were euthanized, and blood and right hind paw tissues were collected for subsequent analyses.

### Measurement of Paw Edema in Mice

Right hind paw thickness was measured at 0, 2, 4, and 6 h after intraplantar injection using a digital caliper. The change in paw thickness (Δpaw thickness) was calculated by subtracting the baseline values at 0 h from the value measured at 2, 4, and 6 h.

### Measurement of Evans Blue Dye Extravasation in Mice

To evaluate vascular leakage, Evans blue dye extravasation was assessed with slight modifications to a previously described method [24]. At 6 h after intraplantar injection of λ-carrageenan or PBS, mice were intravenously injected with 100 μL of 2% (w/v) Evans blue solution via the tail vein. Thirty minutes after Evans blue injection, the mice were euthanized, and paw tissues were collected. The tissues were dried at 65°C for 24 h and weighed. Evans blue was extracted from the dried tissues by incubation in 500 μL of formamide at 55°C for 24 h. The absorbance of the resulting supernatant was measured at 620 nm using a microplate reader. The amount of extravasated Evans blue was quantified using a standard curve and normalized to the dried tissue weight. Results were expressed as μg Evans blue/g dry tissue.

### Hematoxylin and Eosin Staining and Histopathological Analysis in Mice

Right hind paw tissues were fixed in 10% neutral buffered formalin (Sigma-Aldrich), decalcified in 10% EDTA, embedded in paraffin, sectioned at 5 μm, and stained with hematoxylin and eosin (H&E). Histopathological images were acquired using a Zeiss Axiovert 100M microscope (Carl Zeiss, Germany). Subplantar tissue thickness (μm) was quantified using ImageJ software (National Institutes of Health, USA). The severity of inflammatory cell infiltration was evaluated in a blinded manner using an ordinal scale from 0 (normal) to 5 (severe).

### Measurement of Serum Biomarkers in Mice

Blood samples were centrifuged at 2,000 × g for 15 min at 4ºC to obtain serum. Serum biomarker analyses were performed by LABISKOMA (Republic of Korea). The serum concentrations of TNF-α, keratinocyte-derived chemokine (KC), monocyte chemoattractant protein-1 (MCP-1), and soluble intercellular adhesion molecule-1 (sICAM-1) were measured using a Mouse Cytokine/Chemokine/Growth Factor Expanded 7-Plex Magnetic Bead Panel (Cat. No. MCYT1-190K-07; Merck Millipore, USA) according to the manufacturer’s instructions. The serum concentration of soluble vascular cell adhesion molecule-1 (sVCAM-1) was measured using a Mouse VCAM-1/CD106 Quantikine ELISA Kit (Cat. No. MVC00; R&D Systems, USA).

### Preparation and Quantification of HY7801-Derived Extracellular Vesicles and Cell Viability Assay

The HY7801-derived EV preparation used in the present study was from the same batch previously used and characterized in our previous study [23], in which particle size distribution and particle concentration were characterized by nanoparticle tracking analysis (NTA). No additional NTA characterization was performed for the aliquots used in the present study. The total protein concentration of the EV preparations was quantified using a bicinchoninic acid (BCA) protein assay, and EV treatment concentrations were standardized based on total protein content for *in vitro* experiments.

To evaluate cytotoxicity, HUVECs were seeded in 96-well plates and cultured in growth medium until approximately 80% confluence was reached. The cells were then treated with HY7801 EVs for 24 h. Following incubation, 0.5 mg/mL MTT solution was added to each well, and the cells were incubated for an additional 4 h at 37°C. After incubation, the supernatant was removed, and 150 μL of DMSO was added to each well to dissolve the resulting formazan crystals. Absorbance was measured at 540 nm using a BioTek Synergy HTX Multimode Reader. Cell viability was expressed as a percentage relative to the non-treated (NT) group. For subsequent gene expression analysis, HUVECs were pretreated with HY7801 EVs for 24 h, followed by stimulation with 10 ng/mL TNF-α for 6 h. Following incubation, the mRNA expression levels of target genes were analyzed using qRT-PCR.

### Quantitative Real-Time PCR

Total RNA was extracted from RAW 264.7 cells, HUVECs, and mouse hind paw tissues using the Easy-spin Total RNA Extraction Kit (iNtRON Biotechnology, Republic of Korea) according to the manufacturer’s instructions. cDNA was synthesized using the Omniscript Reverse Transcription Kit (Qiagen, Germany). Quantitative real-time PCR (qRT-PCR) was performed using TaqMan^TM^ Gene Expression Assays (Applied Biosystems, USA).

The target genes analyzed were as follows: for RAW 264.7 cells, *iNOS* (Mm00440502_m1); for HUVECs, *eNOS* (Hs01574665_m1), *ICAM1* (Hs00164932_m1), *VCAM1* (Hs01003372_m1), *SELE* (Hs00174057_m1), *CXCL8* (Hs00174103_m1), *TJP1* (Hs01551871_m1), and *CLDN5* (Hs00533949_s1); for mouse paw tissues, *Tjp1* (Mm01320638_m1) and *Cldn5* (Mm00727012_s1).

Relative gene expression was calculated using the 2^-ΔΔCt^ method and normalized to *Gapdh* (Mm99999915_g1) for mouse-derived samples (RAW 264.7 cells and paw tissues) and *GAPDH* (Hs02786624_g1) for HUVECs. Samples for which no Ct value was obtained within 40 amplification cycles were considered below the detection limit. Because *iNOS* expression in the non-treated RAW 264.7 cells was below the detection limit, the LPS-treated group was used as the calibrator (assigned a relative expression value of 1.00). For the other target genes, relative expression was calculated using the corresponding non-treated or normal group as the calibrator.

### Statistical Analysis

Statistical analyses were performed using GraphPad Prism version 10 (GraphPad Software, USA). Data are presented as the mean ± standard deviation (SD). For single-time-point continuous outcomes, differences among groups were analyzed using one-way analysis of variance (ANOVA) followed by Tukey’s multiple-comparisons test. Changes in paw thickness over time were analyzed using two-way repeated-measures ANOVA followed by Dunnett’s multiple-comparisons test for comparisons with the CRG group at each time point. Histopathological inflammation scores were analyzed using the Kruskal-Wallis test followed by Dunn’s post hoc test. Outliers were identified and excluded using the 1.5 × interquartile range (IQR) criterion. Accordingly, the final sample size ranged from 7 to 8 mice per group for biochemical, molecular, and histopathological measurements and from 3 to 4 mice per group for the Evans blue extravasation assay. Statistical significance was set at *p* < 0.05.

## Results

### HY7801 Differentially Regulates NO-Related Responses in LPS-Stimulated Macrophages and TNF-α-Stimulated HUVECs

To evaluate the effects of HY7801 on NO-related responses in inflammatory and endothelial cell models, nitrite accumulation and NOS-associated gene expression were examined in LPS-stimulated RAW 264.7 macrophages and TNF-α-stimulated HUVECs, respectively. In RAW 264.7 cells, LPS stimulation markedly increased nitrite levels to 46.09 μM compared with 1.64 μM in the NT group (*p* < 0.001). Treatment with HY7801 significantly reduced LPS-induced nitrite accumulation to 29.35 μM and 28.45 μM at 1 × 10^6^ and 1 × 10^7^ CFU/mL, respectively (*p* < 0.001; Fig. 1A). Consistent with the changes in nitrite accumulation, *iNOS* mRNA expression in the NT group was below the detection limit under the assay conditions, with no Ct value obtained within 40 amplification cycles. In contrast, *iNOS* was detected following LPS stimulation. Accordingly, the LPS-treated group was used as the calibrator and assigned a relative expression value of 1.00. HY7801 treatment significantly downregulated *iNOS* mRNA expression to 0.88-fold at 1 × 10^6^ CFU/mL (*p* < 0.01) and 0.76-fold at 1 × 10^7^ CFU/mL (*p* < 0.001) relative to the LPS-treated group (Fig. 1B).

In HUVECs, TNF-α stimulation significantly decreased nitrite levels from 1.53 μM in the NT group to 1.11 μM (*p* < 0.01). HY7801 treatment significantly increased nitrite levels to 1.50 μM at 1 × 10^6^ CFU/mL (*p* < 0.01) and 1.92 μM at 1 × 10^7^ CFU/mL (*p* < 0.001; Fig. 1C). TNF-α stimulation also significantly downregulated *eNOS* mRNA expression to 0.75-fold relative to the NT group (*p* < 0.05). HY7801 treatment increased *eNOS* mRNA expression to 0.97-fold and 1.09-fold at 1 × 10^6^ and 1 × 10^7^ CFU/mL, respectively, with a statistically significant increase observed at 1 × 10^7^ CFU/mL compared with the TNF-α-treated group (*p* < 0.01; Fig. 1D).

### HY7801 Downregulates Endothelial Activation-Associated Gene Expression in TNF-α-Stimulated HUVECs

We examined the mRNA expression of the endothelial adhesion molecules *ICAM1*, *VCAM1*, and *SELE* and the inflammatory chemokine *CXCL8* in HUVECs. TNF-α stimulation markedly increased *ICAM1*, *VCAM1*, and *SELE* mRNA expression to 266.71-, 549.26-, and 2647.12-fold, respectively, compared with the NT group (*p* < 0.001). HY7801 significantly reduced *ICAM1* expression to 139.02- and 116.64-fold at 1 × 10^6^ and 1 × 10^7^ CFU/mL, respectively (*p* < 0.001). *VCAM1* expression was reduced to 53.85- and 165.09-fold at the two HY7801 concentrations, respectively (*p* < 0.001). *SELE* expression was reduced to 1290.01-fold (*p* < 0.01) and 300.40-fold (*p* < 0.001) at 1 × 10^6^ and 1 × 10^7^ CFU/mL, respectively (Fig. 2A-2C).

TNF-α stimulation also increased *CXCL8* mRNA expression to 121.66-fold compared with the NT group (*p* < 0.001). HY7801 treatment significantly reduced *CXCL8* expression to 74.69- and 73.58-fold at 1 × 10^6^ and 1 × 10^7^ CFU/mL, respectively (*p* < 0.05; Fig. 2D).

### HY7801 Upregulates Junction-Associated Gene Expression and Attenuates Endothelial Barrier Dysfunction in TNF-α-Stimulated HUVECs

To assess endothelial barrier-associated responses, *TJP1* and *CLDN5* mRNA expression and TEER were measured in TNF-α-stimulated HUVECs. TNF-α reduced *TJP1* mRNA expression to 0.73-fold relative to the NT group, although this difference was not statistically significant. HY7801 treatment increased *TJP1* expression to 1.05- and 1.26-fold at 1 × 10^6^ and 1 × 10^7^ CFU/mL, respectively, with a significant increase at the higher concentration compared with TNF-α alone (*p* < 0.01; Fig. 3A). TNF-α significantly reduced *CLDN5* mRNA expression to 0.75-fold (*p* < 0.05), whereas HY7801 treatment significantly upregulated its expression to 1.38- and 1.49-fold at 1 × 10^6^ and 1 × 10^7^ CFU/mL, respectively (*p* < 0.001; Fig. 3B).

Consistent with these findings, TNF-α stimulation markedly reduced TEER from 126.09 Ω·cm^2^ in the NT group to 59.15 Ω·cm^2^ (*p* < 0.001). However, HY7801 treatment partially attenuated this decline, increasing TEER to 83.28 Ω·cm^2^ (*p* < 0.05) and 87.17 Ω·cm^2^ (*p* < 0.01) at 1 × 10^6^ and 1 × 10^7^ CFU/mL, respectively (Fig. 3C). TEER nevertheless remained below the NT group value.

### HY7801 Attenuates λ-Carrageenan-Induced Paw Edema in a Mouse Model

To evaluate the *in vivo* anti-edematous effect of HY7801, paw swelling was monitored for 6 h following intraplantar λ-carrageenan injection. Δpaw thickness was calculated by subtracting the baseline measurement obtained immediately before carrageenan injection.

λ-Carrageenan injection markedly increased Δpaw thickness at 2, 4, and 6 h compared with the NOR group (*p* < 0.001), confirming the development of acute paw edema. At 2 h after carrageenan injection, Δpaw thickness reached 1.03 mm in the CRG group. The HY7801H group showed a significantly smaller increase in paw thickness of 0.66 mm compared with the CRG group (*p* < 0.001). Paw swelling remained elevated at the later time points. At 6 h after carrageenan injection, Δpaw thickness was 2.16 mm in the CRG group. The DCF group showed a significant reduction in Δpaw thickness to 1.83 mm (*p* < 0.01), while the HY7801H group showed a significant reduction to 1.81 mm compared with the CRG group (*p* < 0.001; Fig. 4A).

Representative macroscopic images of the hind paws were consistent with the quantitative measurements and showed visibly reduced paw swelling in the HY7801-treated groups compared with the CRG group (Fig. 4B).

### HY7801 Reduces Evans Blue Extravasation in Paw Tissue

To assess vascular leakage during carrageenan-induced paw inflammation, Evans blue accumulation was evaluated in paw tissue. Representative images showed pronounced blue dye accumulation in the paw of the CRG group compared with the NOR group, whereas visibly less dye accumulation was observed in the HY7801-treated groups (Fig. 5A).

Consistent with the representative images, quantitative analysis showed that λ-carrageenan injection markedly increased Evans blue accumulation to 164.61 μg/g compared with 39.70 μg/g in the NOR group (*p* < 0.001). Diclofenac treatment reduced Evans blue accumulation to 128.07 μg/g; however, this reduction was not statistically significant compared with the CRG group. In contrast, HY7801 significantly reduced Evans blue accumulation at both administered doses, the levels decreasing to 79.90 μg/g in the HY7801L group (*p* < 0.01) and 75.85 μg/g in the HY7801H group (*p* < 0.001) compared with the CRG group (Fig. 5B). These findings demonstrate that HY7801 administration was associated with reduced Evans blue extravasation in λ-carrageenan-inflamed paw tissue.

### HY7801 Attenuates λ-Carrageenan-Induced Histopathological Changes

Histopathological changes in the hind paw were examined by H&E staining. Representative tissue sections from the CRG group showed marked subplantar tissue swelling and inflammatory cell infiltration compared with the NOR group. These histopathological changes appeared less pronounced in the diclofenac- and HY7801-treated groups (Fig. 6A).

Quantitative analysis showed that subplantar tissue thickness increased from 349.19 μm in the NOR group to 666.02 μm in the CRG group (*p* < 0.001). HY7801 significantly reduced tissue thickness to 523.54 μm in the HY7801L group (*p* < 0.05) and 477.89 μm in the HY7801H group (*p* < 0.001) compared with the CRG group (Fig. 6B).

The histopathological inflammation score was also significantly elevated by λ-carrageenan, from 1.17 in the NOR group to 3.50 in the CRG group (*p* < 0.01). Although lower inflammation scores were observed in the diclofenac- and HY7801-treated groups, these reductions did not reach statistical significance compared with the CRG group (Fig. 6C). Thus, HY7801 significantly reduced λ-carrageenan-induced tissue thickening, whereas the decrease in histopathological inflammation score showed a non-significant trend.

### HY7801 Increases Tight Junction-Related Gene Expression in a λ-Carrageenan-Induced Paw Edema Model

To determine whether the reduction in vascular leakage was accompanied by changes in tight junction-related gene expression *in vivo*, *Tjp1* and *Cldn5* mRNA levels were measured in whole hind paw tissue. Carrageenan injection significantly reduced *Tjp1* mRNA expression to 0.14-fold compared with the NOR group (*p* < 0.001). HY7801 significantly increased *Tjp1* expression relative to the CRG group, reaching 0.38-fold in the HY7801L group (*p* < 0.01) and 0.34-fold in the HY7801H group (*p* < 0.05; Fig. 7A). Although both HY7801 doses significantly increased *Tjp1* expression relative to the CRG group, the expression levels remained below those observed in the NOR group.

Carrageenan also significantly reduced *Cldn5* mRNA expression to 0.54-fold relative to the NOR group (*p* < 0.05). Diclofenac treatment increased *Cldn5* expression to 0.99-fold relative to the NOR group, representing a significant increase compared with the CRG group (*p* < 0.05). HY7801 increased *Cldn5* expression to 0.72-fold in the HY7801L group and 1.10-fold in the HY7801H group. A statistically significant increase compared with the CRG group was observed in the HY7801H group (*p* < 0.01; Fig. 7B).

### HY7801 Attenuates Circulating Inflammatory Mediators in a λ-Carrageenan-Induced Paw Edema Model

To evaluate changes in circulating inflammatory mediators following carrageenan injection, serum concentrations of the pro-inflammatory cytokine TNF-α, the chemokines KC and MCP-1, and the soluble cell adhesion molecules sICAM-1 and sVCAM-1 were measured. Carrageenan injection significantly increased serum TNF-α concentrations from 61.36 pg/mL in the NOR group to 116.43 pg/mL in the CRG group (*p* < 0.001). HY7801 significantly reduced TNF-α concentrations to 93.48 pg/mL in the HY7801L group and 92.73 pg/mL in the HY7801H group compared with the CRG group (*p* < 0.05; Fig. 7C).

Serum KC concentrations were markedly increased by λ-carrageenan, from 531.20 pg/mL in the NOR group to 6054.84 pg/mL in the CRG group (*p* < 0.001). HY7801 significantly reduced KC concentrations to 3695.83 pg/mL in the HY7801L group (*p* < 0.01) and 2426.76 pg/mL in the HY7801H group (*p* < 0.001) compared with the CRG group (Fig. 7D).

Serum MCP-1 concentrations also increased following λ-carrageenan injection, from 354.75 pg/mL in the NOR group to 469.41 pg/mL in the CRG group (*p* < 0.01). HY7801 treatment significantly reduced MCP-1 concentrations in both HY7801-treated groups compared with the CRG group (*p* < 0.05; Fig. 7E).

Among the soluble adhesion molecules examined, serum sICAM-1 concentrations increased from 360.51 ng/mL in the NOR group to 392.46 ng/mL in the CRG group (*p* < 0.01). Diclofenac significantly reduced sICAM-1 to 369.93 ng/mL (*p* < 0.05), while HY7801H similarly reduced sICAM-1 to 370.45 ng/mL compared with the CRG group (*p* < 0.05; Fig. 7F). In contrast, serum sVCAM-1 concentrations did not differ significantly among the experimental groups (Fig. 7G).

### HY7801 EVs Enhance Tight Junction-Associated Gene Expression and Attenuate Adhesion Molecule Expression in TNF-α-Stimulated HUVECs

HY7801 EVs showed no substantial cytotoxicity at concentrations up to 10 μg/mL, with cell viability remaining at 96.01% (Fig. 8A). In contrast, treatment with 100 μg/mL significantly reduced cell viability to 78.99% (*p* < 0.001). Therefore, HY7801 EVs at concentrations of 1 and 10 μg/mL were used for subsequent experiments.

TNF-α stimulation reduced the mRNA expression of tight junction-associated genes *TJP1* and *CLDN5*. *TJP1* expression decreased to 0.65-fold following TNF-α stimulation, whereas treatment with HY7801 EVs significantly increased its expression to 1.82- and 1.39-fold at 1 and 10 μg/mL, respectively (*p* < 0.001; Fig. 8B). *CLDN5* expression was significantly reduced to 0.79-fold following TNF-α stimulation compared with the NT group (*p* < 0.01), whereas treatment with 10 μg/mL HY7801 EVs significantly increased its expression to 1.17-fold (*p* < 0.001; Fig. 8C).

TNF-α stimulation markedly increased the mRNA expression of the endothelial adhesion molecules *ICAM1*, *VCAM1*, and *SELE*. *ICAM1* expression increased to 311.78-fold following TNF-α stimulation (*p* < 0.001), whereas treatment with HY7801 EVs significantly reduced its expression to 168.18- and 138.84-fold at 1 and 10 μg/mL, respectively (*p* < 0.001; Fig. 8D). Similarly, *VCAM1* expression was significantly increased to 1338.19-fold following TNF-α stimulation (*p* < 0.001), and treatment with 10 μg/mL HY7801 EVs significantly reduced its expression to 733.54-fold (*p* < 0.05; Fig. 8E). *SELE* expression was also increased to 2716.94-fold by TNF-α stimulation (*p* < 0.001), whereas treatment with HY7801 EVs significantly reduced its expression to 1760.44-fold at 1 μg/mL (*p* < 0.05) and 1124.94-fold at 10 μg/mL (*p* < 0.001; Fig. 8F).

## Discussion

Acute inflammatory edema arises from the interplay between excessive inflammatory activation and disruption of the microvascular endothelial barrier, leading to plasma protein and fluid extravasation and tissue swelling [3]. Although conventional anti-inflammatory therapies primarily suppress inflammatory mediator production, endothelial hyperpermeability represents a distinct pathological component that may benefit from complementary barrier-directed approaches [16, 25]. In the present study, *Lactobacillus helveticus* HY7801 attenuated carrageenan-induced paw edema and vascular leakage while modulating inflammatory and endothelial responses. Across complementary cellular and animal models, its anti-edematous activity was associated with reduced inflammatory activation and preservation of endothelial barrier-associated responses. These findings suggest that HY7801 may exert coordinated anti-inflammatory and vascular barrier-supporting effects.

A notable finding of the present study was the differential regulation of NO-related responses in macrophages and endothelial cells. During acute inflammation, high-output NO production by iNOS-expressing macrophages can amplify inflammatory and nitrosative stress, whereas basal eNOS-derived NO contributes to vascular homeostasis and endothelial junctional integrity [9, 26]. However, the vascular effects of NO are highly context dependent and vary according to the NOS isoform involved, the amount and duration of NO production, and the surrounding redox environment; under certain inflammatory conditions, stimulus-induced eNOS activation may also contribute to vascular hyperpermeability [27, 28]. HY7801 reduced LPS-induced nitrite accumulation and *iNOS* expression in macrophages while increasing nitrite accumulation and *eNOS* expression in TNF-α-stimulated HUVECs. This reciprocal response suggests that HY7801 may modulate NO-related responses in a cell context-dependent manner, potentially limiting inflammatory edema by suppressing excessive immune-cell activation while supporting endothelial function.

The anti-inflammatory activity of HY7801 was further supported by reduced *ICAM1*, *VCAM1*, *SELE*, and *CXCL8* mRNA expression in TNF-α-stimulated HUVECs. The molecules encoded by these genes act at complementary stages of leukocyte recruitment: E-selectin mediates the initial capture and rolling of circulating leukocytes, ICAM-1 and VCAM-1 support firm adhesion and transendothelial migration, and *CXCL8* provides a potent chemotactic signal for neutrophil recruitment [29-31]. The coordinated reduction in these transcripts suggests that HY7801 may attenuate the endothelial transcriptional program associated with leukocyte recruitment and local inflammatory amplification. This effect may complement the modulation of macrophage NO-related responses by reducing signals associated with immune-endothelial inflammatory crosstalk.

Nevertheless, these findings should be interpreted within the scope of the measurements performed. The endothelial NO response was assessed using total nitrite accumulation and *eNOS* mRNA expression, without direct evaluation of eNOS protein abundance, phosphorylation, or enzymatic activity. Similarly, reductions in adhesion molecule and chemokine transcripts do not necessarily indicate corresponding changes in protein abundance or surface expression and therefore do not establish a functional reduction in leukocyte adhesion [32]. Thus, the present results support an association between HY7801 treatment and coordinated modulation of macrophage and endothelial inflammatory responses, whereas the specific signaling mechanisms and functional consequences for leukocyte-endothelial interactions require further investigation.

HY7801 also attenuated TNF-α-induced impairment of endothelial monolayer resistance, providing functional evidence for its vascular barrier-supporting effects. TNF-α markedly reduced TEER, whereas HY7801 significantly attenuated this decline. This partial recovery of TEER was accompanied by increased *TJP1* and *CLDN5* mRNA expression, which encode ZO-1 and claudin-5, respectively, two tight junction-associated proteins involved in the regulation of endothelial paracellular permeability [33, 34]. Together, these findings suggest that HY7801 may support endothelial barrier homeostasis by promoting junction-associated responses during inflammation. However, TEER remained below that of untreated cells, indicating that recovery of barrier function was partial rather than complete. Moreover, because the molecular analyses were limited to mRNA expression, it remains unclear whether HY7801 preserves junctional protein abundance, membrane localization, or structural continuity.

We next examined whether the *in vitro* endothelial and inflammatory responses were accompanied by reduced vascular leakage and edema formation *in vivo*. The λ-carrageenan-induced paw edema model is a well-established model of acute localized inflammation characterized by time-dependent edema formation, inflammatory cell recruitment, and increased inflammatory mediator production [35, 36]. HY7801 significantly attenuated paw swelling and histological tissue thickening and markedly reduced Evans blue accumulation in inflamed paw tissue. The accompanying increase in vascular permeability and inflammatory exudation makes this model suitable for evaluating both anti-inflammatory and anti-edematous responses [24]. Because Evans blue binds circulating albumin, its extravasation into tissue is widely used as an index of vascular hyperpermeability and plasma protein leakage [37]. Accordingly, the pronounced reduction in Evans blue accumulation suggests that the anti-edematous activity of HY7801 was associated not only with attenuation of inflammatory responses but also with reduced vascular leakage.

Interestingly, diclofenac significantly reduced paw swelling but did not significantly decrease Evans blue accumulation under the present experimental conditions, whereas HY7801 significantly reduced both outcomes. This differential pattern may reflect distinct effects on components of inflammatory edema and vascular leakage; however, the study was not designed to directly compare the efficacy or mechanisms of the two interventions. The lack of a significant effect of diclofenac on Evans blue accumulation may also have been influenced by the dose, treatment schedule, assay timing, or experimental variability.

The *in vivo* changes in junction-associated gene expression were broadly consistent with the reduction in vascular leakage. Carrageenan decreased *Tjp1* and *Cldn5* mRNA expression in whole paw tissue, whereas HY7801 partially restored both transcripts. The increase in *Cldn5* mRNA in the HY7801H group is of particular interest because claudin-5 is a major endothelial tight junction protein that contributes to the regulation of paracellular permeability and vascular barrier integrity [34, 38]. These findings support an association between HY7801 treatment and improved barrier-associated molecular responses in inflamed tissue. However, because the analyses were performed using whole paw tissue, the observed changes cannot be attributed specifically to endothelial cells.

HY7801 also reduced the serum concentrations of several circulating inflammatory mediators, including TNF-α, KC, MCP-1, and sICAM-1. These findings suggest that the local protective effects observed in the inflamed paw were accompanied by attenuation of selected circulating inflammatory responses. TNF-α contributes to inflammatory macrophage-endothelial crosstalk and endothelial dysfunction, whereas KC and MCP-1 are important chemotactic mediators of neutrophil and monocyte recruitment, respectively [39-41]. Their reduction is consistent with the reduced tissue thickening and the numerically lower inflammation scores observed in HY7801-treated mice. The decrease in circulating sICAM-1 further supports an association between HY7801 treatment and reduced inflammatory activation; however, sICAM-1 expression is not restricted to endothelial cells and is also inducible in epithelial and immune cells during inflammation [42]. Circulating sICAM-1 should therefore not be interpreted as an endothelial-specific marker. Moreover, the absence of a significant change in sVCAM-1 indicates that the effect of HY7801 was not uniform across soluble adhesion molecules. Differences in cellular origin, release kinetics, and responsiveness to the inflammatory stimulus may have contributed to the distinct patterns observed for sICAM-1 and sVCAM-1.

Given the endothelial barrier-associated effects observed with HY7801 *in vitro* and the reduction in vascular leakage *in vivo*, we further explored HY7801 EVs as candidate bacterial-derived bioactive components capable of modulating endothelial responses. HY7801-derived EVs have previously been characterized by NTA, with a mean particle size of approximately 150 nm, supporting the presence of nanosized vesicular particles released by this strain [23]. A previous study also demonstrated the biological activity of HY7801 EVs in host cells, providing evidence that these vesicles can mediate strain-derived cellular responses. In the present study, HY7801 EVs increased the mRNA expression of *TJP1* and *CLDN5* while reducing TNF-α-induced *ICAM1*, *VCAM1*, and *SELE* expression in HUVECs at concentrations that did not adversely affect cell viability. These findings extend the previously reported biological activity of HY7801 EVs by demonstrating their potential to modulate endothelial junction-associated and inflammatory responses. Bacterial EVs can act as carriers of biologically active molecules and facilitate bacterial-host communication, providing a plausible mechanism by which bacterial-derived factors may influence host cellular responses [43]. Nevertheless, the present EV analysis was limited to transcriptional endpoints and did not directly assess corresponding protein abundance, and the specific EV-associated molecules responsible for these effects remain unknown.

Taken together, the *in vitro* and *in vivo* findings support an integrated model in which HY7801 is associated with modulation of both inflammatory and endothelial components of acute inflammatory edema. Several limitations should, however, be considered. First, many mechanistic endpoints were assessed at the transcriptional level, and additional protein- and pathway-level analyses are required to confirm the molecular mechanisms underlying the effects of HY7801. Second, HUVECs are a large-vessel endothelial model and do not directly represent the microvascular endothelium of the paw; similarly, whole-paw tissue does not permit endothelial-specific attribution of *Tjp1* or *Cldn5* expression. Validation in microvascular endothelial cells and endothelial-specific tissue analyses is therefore warranted. Although HY7801 EVs reproduced junction-associated and anti-inflammatory transcriptional responses in HUVECs, these findings do not establish EVs as the sole or principal mediators of the effects of whole HY7801. Other bacterial cellular components, secreted factors, and host-mediated signaling pathways may also contribute to the observed effects. Further studies are therefore needed to identify the bioactive constituents of HY7801 EVs and determine their functional contribution to endothelial barrier regulation.

In conclusion, *L. helveticus* HY7801 attenuated carrageenan-induced paw edema and vascular leakage while suppressing selected inflammatory responses and improving endothelial barrier-associated responses. These effects were associated with coordinated modulation of NO-related responses, endothelial activation, and junction-associated barrier responses across complementary cellular and animal models. Furthermore, HY7801 EVs enhanced tight junction-associated transcriptional responses and attenuated adhesion molecule expression *in vitro*, supporting a potential role for HY7801 EVs as bacterially derived mediators of endothelial junction-associated and inflammatory responses. Collectively, the findings support the potential of HY7801 to modulate inflammation-associated vascular leakage and tissue edema. Further studies are required to characterize the bioactive constituents of HY7801 EVs, determine their functional contribution to endothelial barrier regulation, and validate the vascular barrier-supporting effects of HY7801 in additional models of inflammatory microvascular dysfunction.

## Figures and Tables

**Fig. 1:**
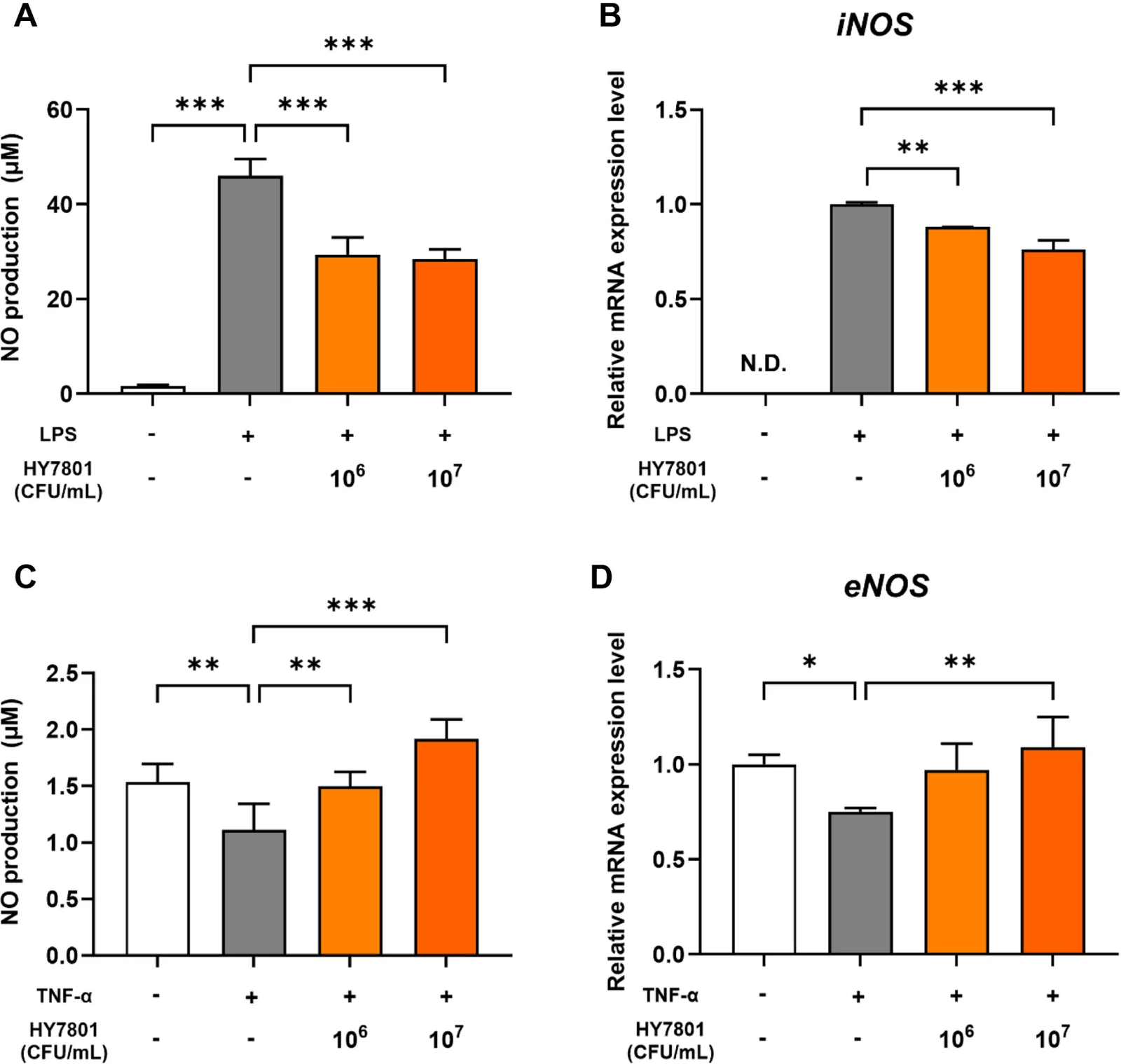
Differential effects of HY7801 on nitric oxide (NO) production and nitric oxide synthase (NOS) mRNA expression in LPS-stimulated RAW 264.7 macrophages and TNF-α-stimulated HUVECs. (**A**) NO production and (**B**) *iNOS* mRNA expression in LPS-stimulated RAW 264.7 cells. *iNOS* expression in the NT group was below the detection limit and was therefore not included in the statistical comparisons (N.D., Not detected). (**C**) NO production and (**D**) *eNOS* mRNA expression in TNF-α-stimulated HUVECs. Data are presented as mean ± SD (*n* = 3, independent experiments). *p < 0.05, **p < 0.01, and ***p < 0.001 indicate significant differences between the indicated groups.

**Fig. 2:**
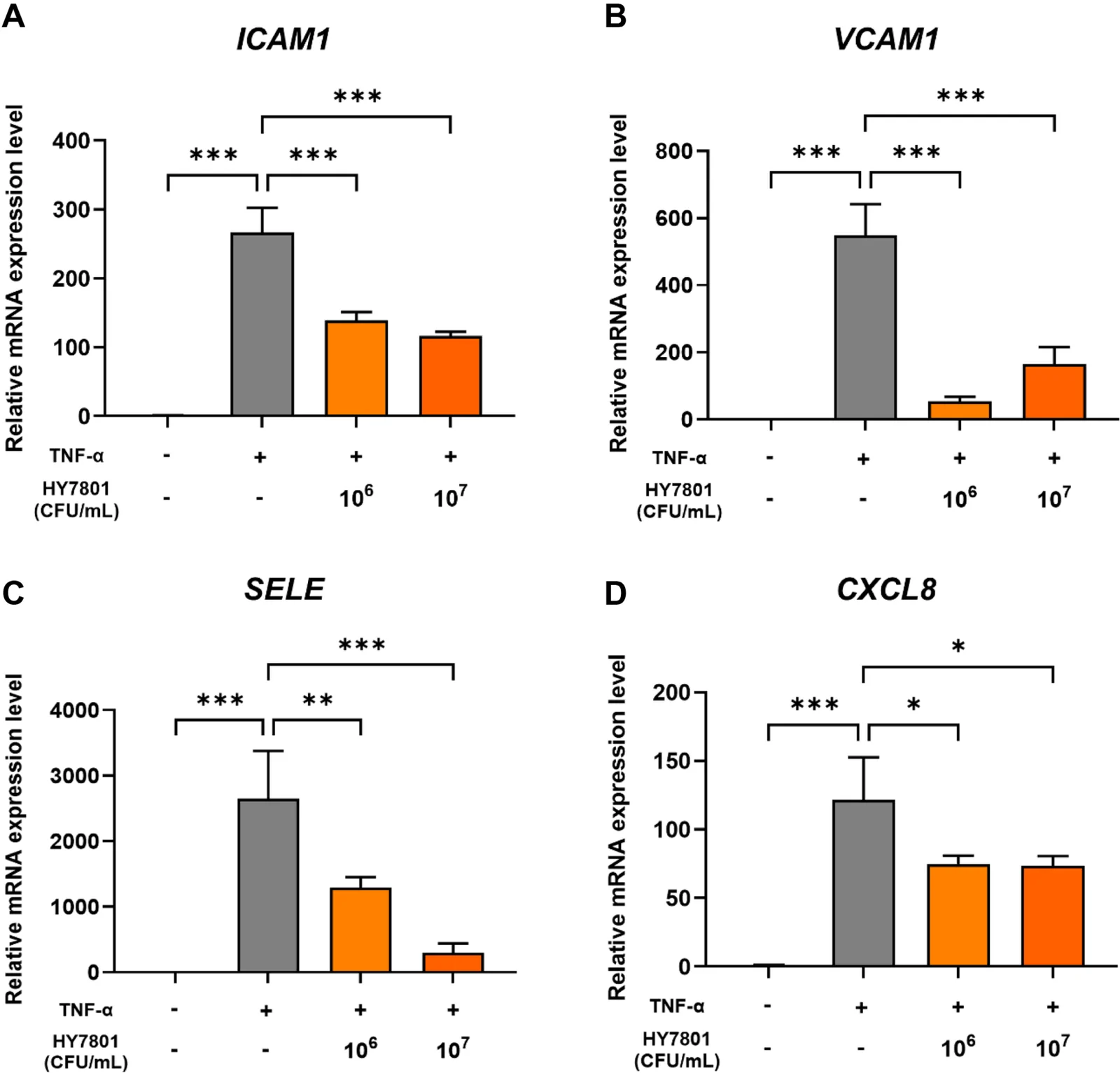
Effects of HY7801 on endothelial activation in TNF-α-stimulated HUVECs. The mRNA expression levels of endothelial adhesion molecules (**A**) *ICAM1*, (**B**) *VCAM1* and (**C**) *SELE* and the chemokine (**D**) *CXCL8* were determined by qRT-PCR in TNF-α-stimulated HUVECs. Data are presented as mean ± SD (*n* = 3, independent experiments). *p < 0.05, **p < 0.01, and ***p < 0.001 indicate significant differences between the indicated groups.

**Fig. 3:**
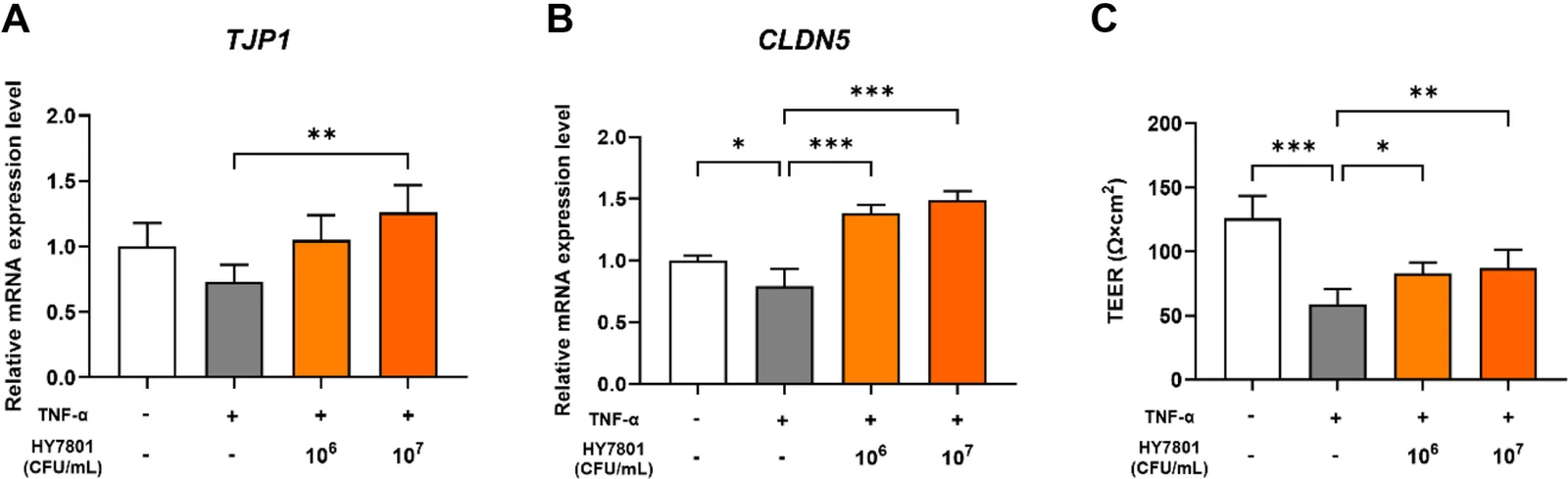
Effects of HY7801 on barrier function in TNF-α-stimulated HUVECs. The mRNA levels of tight junction-associated genes (**A**) *TJP1* and (**B**) *CLDN5* were determined by qRT-PCR in TNF-α-stimulated HUVECs. (**C**) Transendothelial electrical resistance (TEER) was measured to assess endothelial barrier integrity. Data are presented as mean ± SD (*n* = 3, independent experiments). *p < 0.05, **p < 0.01, and ***p < 0.001 indicate significant differences between the indicated groups.

**Fig. 4:**
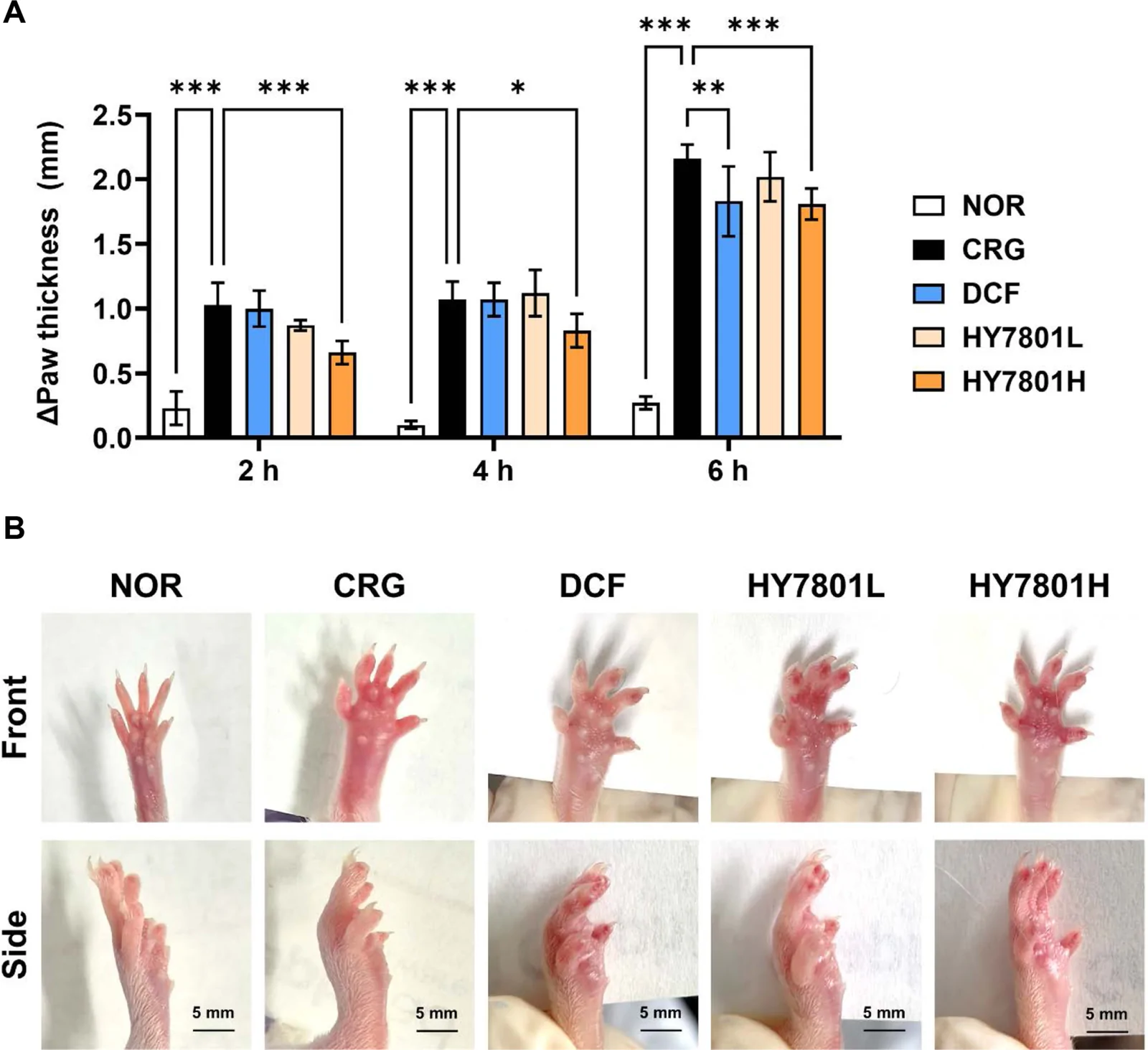
Effects of HY7801 on λ-carrageenan-induced paw edema in mice. (**A**) Changes in paw thickness were measured at 2, 4, and 6 h after λ-carrageenan injection. (**B**) Representative front and side images of the paws at 6 h after λ-carrageenan injection. Scale bar = 5 mm. Data are presented as mean ± SD (*n* = 7–8 mice per group). *p < 0.05, **p < 0.01, and ***p < 0.001 indicate significant differences between the indicated groups.

**Fig. 5:**
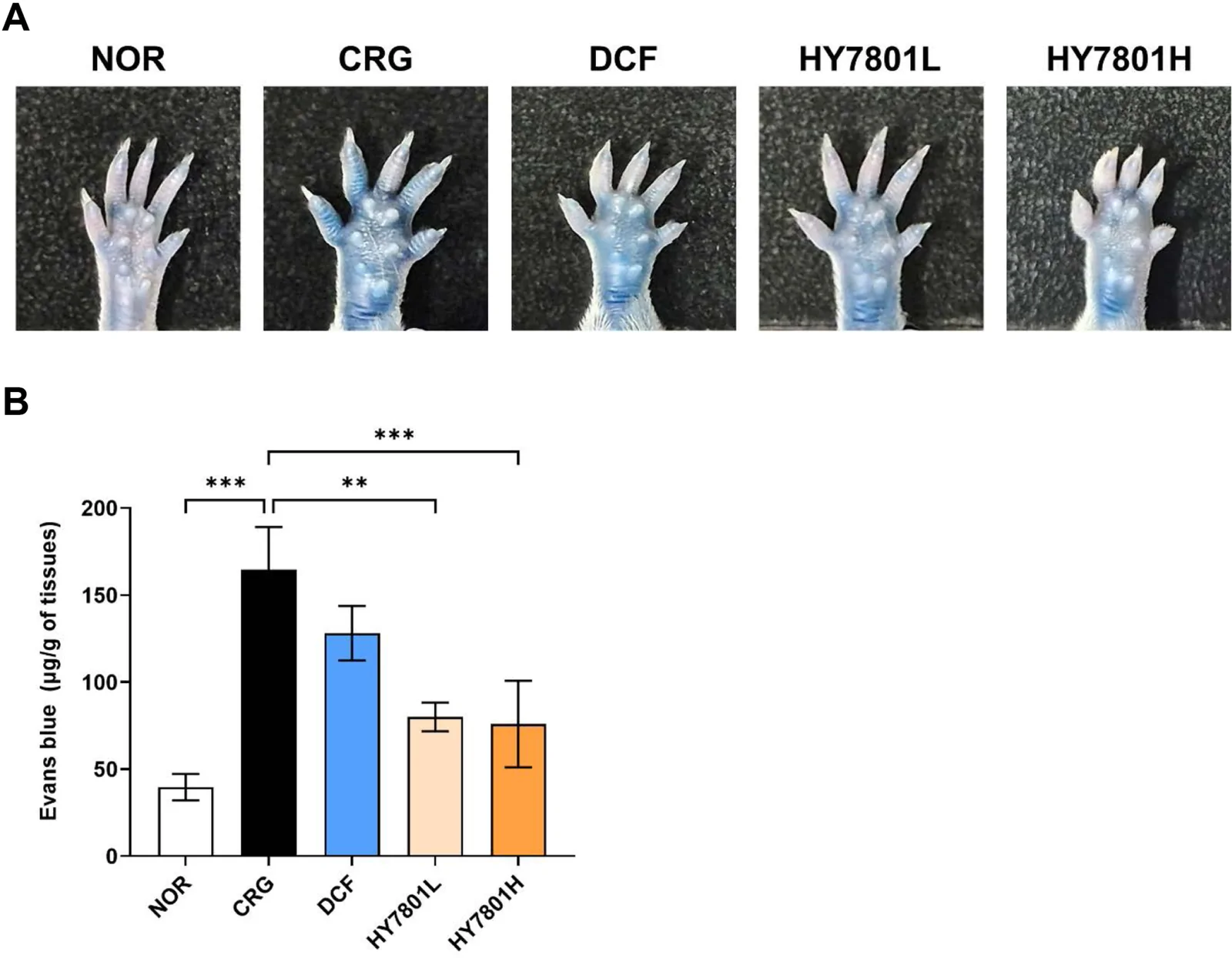
Effects of HY7801 on λ-carrageenan-induced vascular permeability in mice. (**A**) Representative images of Evans blue extravasation in the paws 30 min after Evans blue injection, which was performed at 6 h after λ-carrageenan injection. (**B**) Quantification of extravasated Evans blue in paw tissues (μg/g dry tissue). Data are presented as mean ± SD (*n* = 3–4 mice per group). **p < 0.01, and ***p < 0.001 indicate significant differences between the indicated groups.

**Fig. 6:**
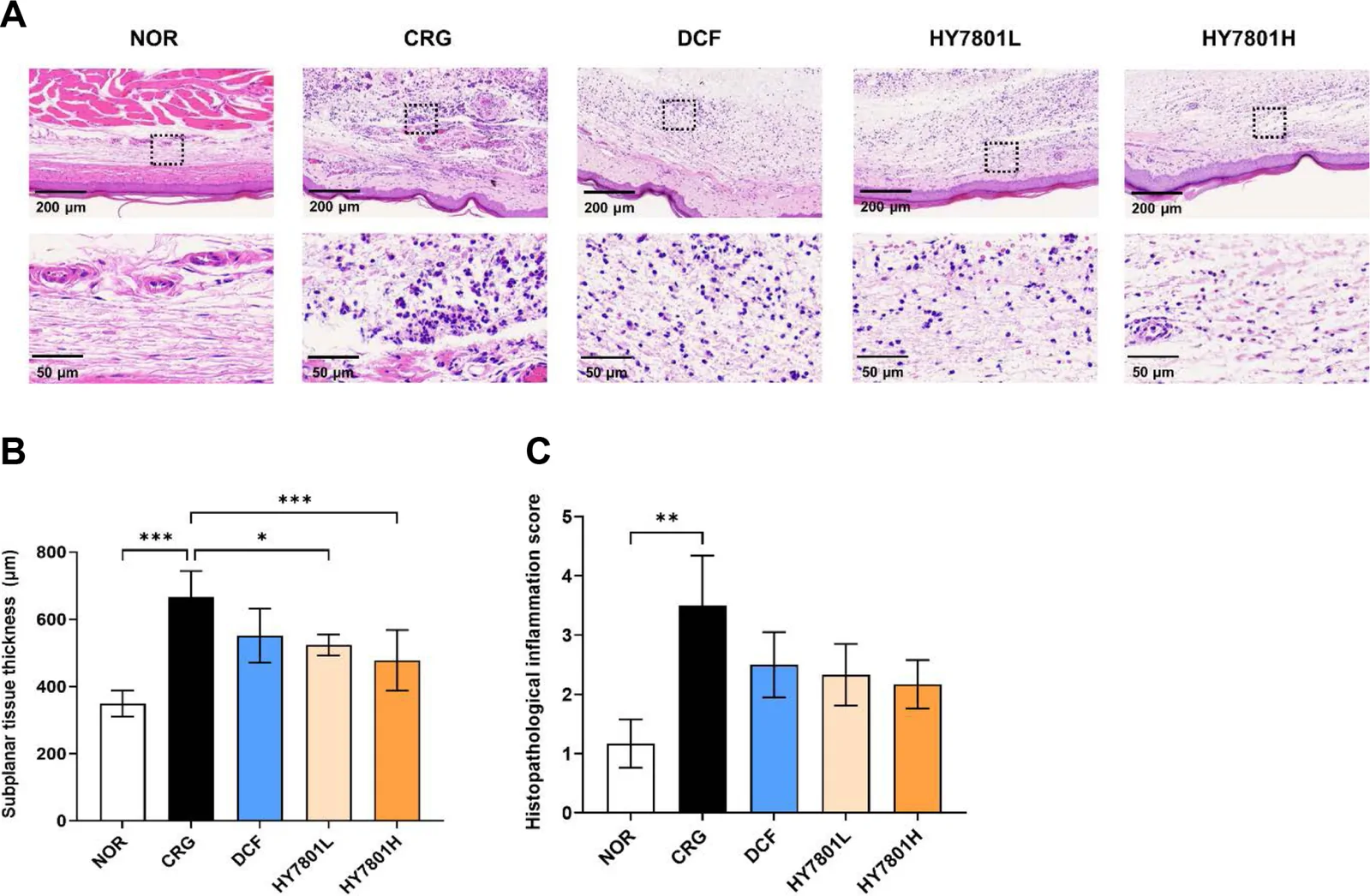
Effects of HY7801 on λ-carrageenan-induced histopathological changes in mouse paw tissues. (A) Representative hematoxylin and eosin (H&E)-stained sections of paw tissues at 6 h after λ-carrageenan injection. Lower panels show magnified views of the boxed areas in the upper panels. Scale bars = 200 μm (upper panels) and 50 μm (lower panels). (B) Quantification of subplantar tissue thickness. (C) Histopathological inflammation scores. Data are presented as mean ± SD (*n* = 8 mice per group). *p < 0.05, **p < 0.01, and ***p < 0.001 indicate significant differences between the indicated groups.

**Fig. 7:**
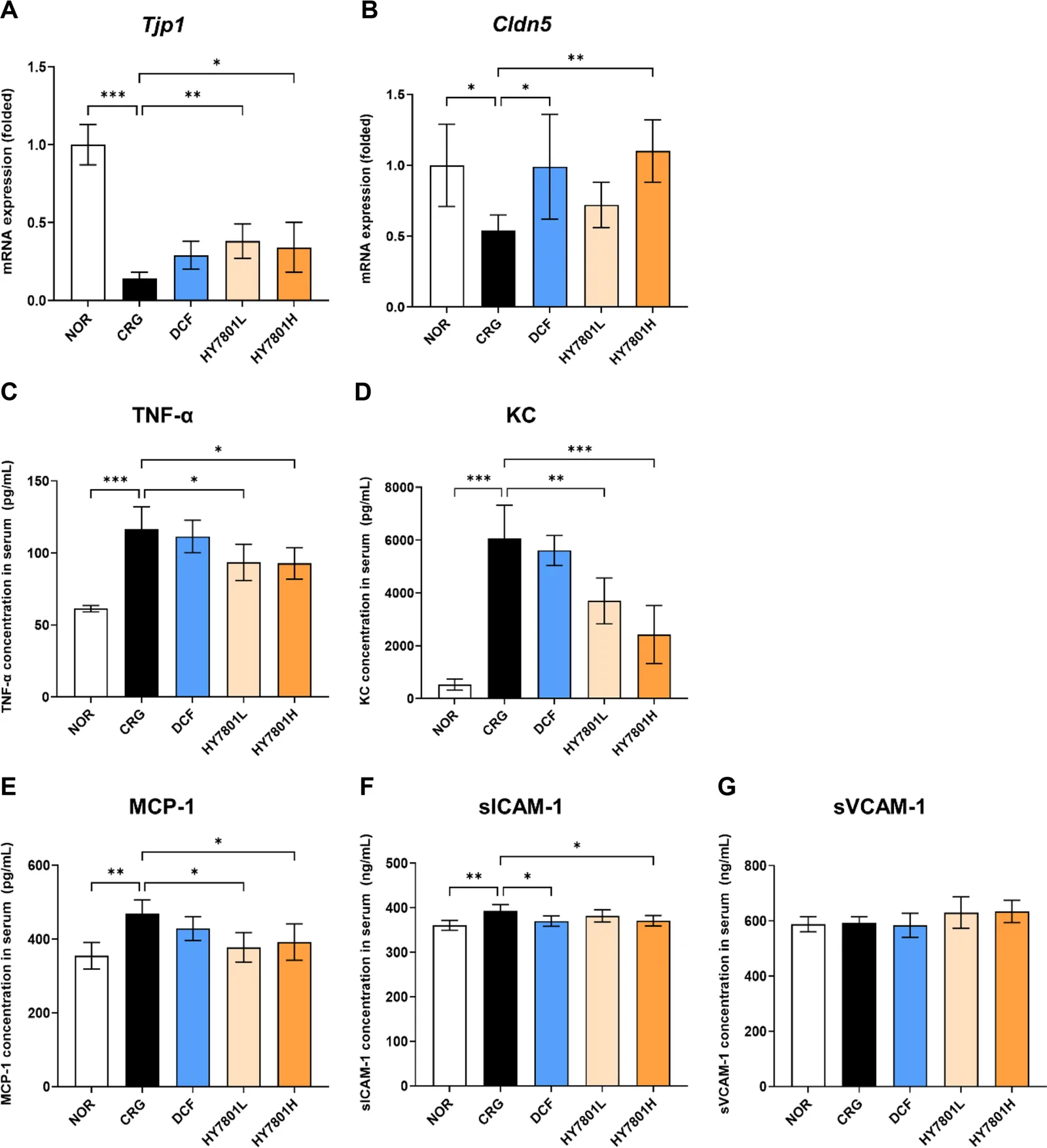
Effects of HY7801 on tight junction-related gene expression and circulating inflammatory markers in λ-carrageenan-induced paw edema in mice. Relative mRNA expression levels of (A) *Tjp1* and (B) *Cldn5* in paw tissues. Serum concentrations of (C) TNF-α, (D) KC, (E) MCP-1, (F) sICAM-1, and (G) sVCAM-1. Data are presented as mean ± SD (*n* = 7–8 mice per group). *p < 0.05, **p < 0.01, and ***p < 0.001 indicate significant differences between the indicated groups.

**Fig. 8:**
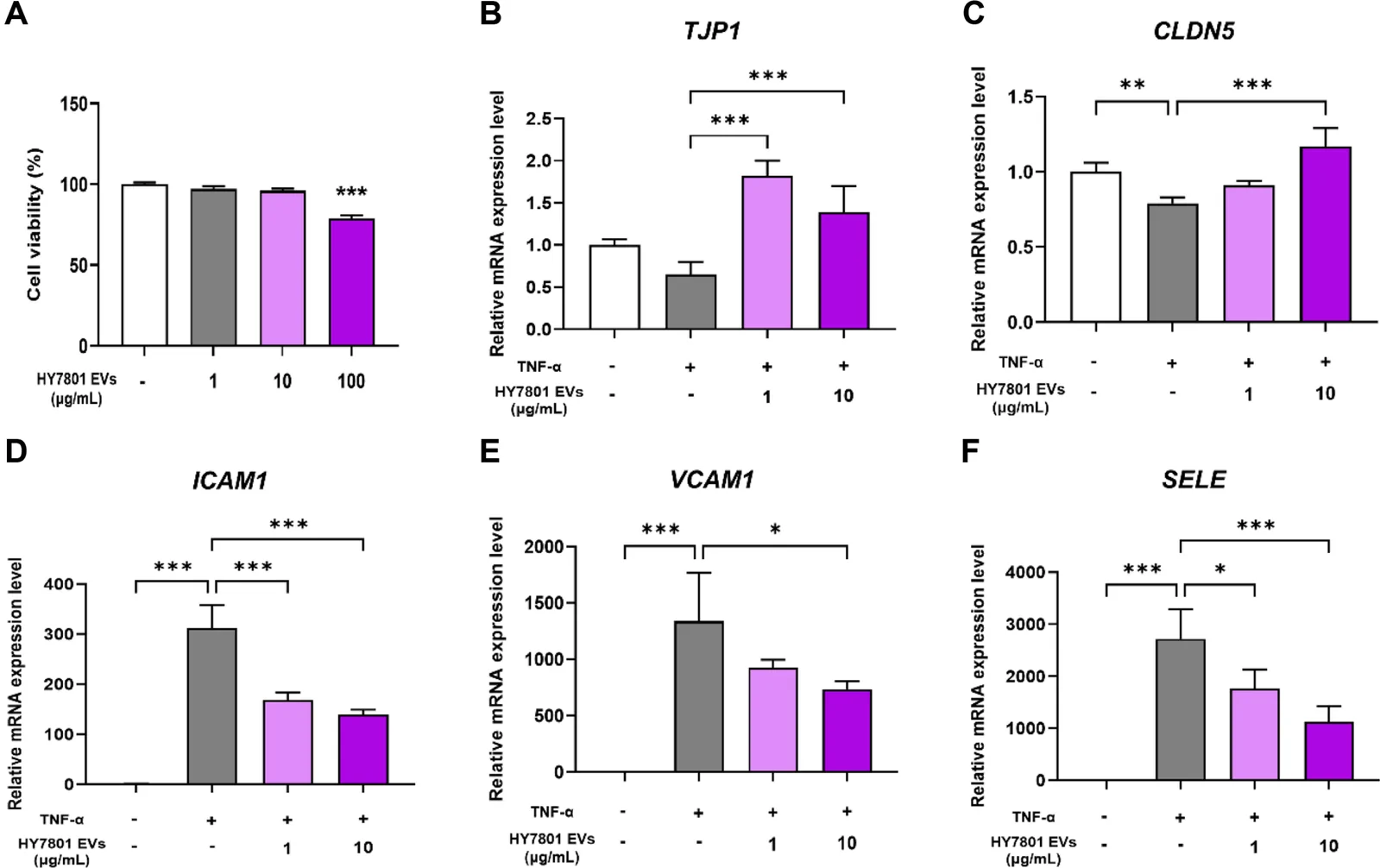
Effects of HY7801-derived extracellular vesicles (HY7801 EVs) on cell viability and endothelial gene expression in TNF-α-stimulated HUVECs. (A) Cell viability following treatment with HY7801 EVs. Relative mRNA expression of tight junction-associated genes (B) *TJP1* and (C) *CLDN5* and endothelial adhesion molecules (D) *ICAM1*, (E) *VCAM1*, and (F) *SELE* in TNF-α-stimulated HUVECs. Data are presented as mean ± SD (*n* = 3, independent experiments). *p < 0.05, **p < 0.01, and ***p < 0.001 indicate significant differences between the indicated groups.
